# Selective control of amino acid metabolism by the GCN2 eIF2 kinase pathway in *Saccharomyces cerevisiae*

**DOI:** 10.1186/1471-2091-11-29

**Published:** 2010-08-04

**Authors:** John M Zaborske, Xiaochen Wu, Ronald C Wek, Tao Pan

**Affiliations:** 1 Department of Biochemistry and Molecular Biology, University of Chicago, Chicago, IL 60637, USA; 2 Department of Biochemistry and Molecular Biology, Indiana University School of Medicine, Indianapolis, Indiana 46202, USA

## Abstract

**Background:**

When eukaryotic cells are deprived of amino acids, uncharged tRNAs accumulate and activate the conserved GCN2 protein kinase. Activated Gcn2p up-regulates the general amino acid control pathway through phosphorylation of the translational initiation factor eIF2. In *Saccharomyces cerevisiae*, Gcn2p is the only kinase that phosphorylates eIF2 to regulate translation through this mechanism. We addressed changes in yeast growth and tRNA aminoacylation, or charging, during amino acid depletion in the presence and absence of *GCN2*. tRNA charging was measured using a microarray technique which simultaneously measures all cytosolic tRNAs. A fully prototrophic strain, and its isogenic *gcn2*Δ counterpart, were used to study depletion for each of the 20 amino acids, with a focus on Trp, Arg, His and Leu, which are metabolically distinct and together provide a good overview on amino acid metabolism.

**Results:**

While the wild-type strain had no observable phenotype upon depletion for any amino acid, the *gcn2*Δ strain showed slow growth in media devoid of only Trp or Arg. Consistent with the growth phenotypes, profiles of genome-wide tRNA charging revealed significant decrease in cognate tRNA charging only in the *gcn2*Δ strain upon depletion for Trp or Arg. In contrast, there was no change in tRNA charging during His and Leu depletion in either the wild-type or *gcn2*Δ strains, consistent with the null effect on growth during loss of these amino acids. We determined that the growth phenotype of Trp depletion is derived from feedback inhibition of aromatic amino acid biosynthesis. By removing Phe and Tyr from the media in addition to Trp, regular growth was restored and tRNA^Trp ^charging no longer decreased. The growth phenotype of Arg depletion is derived from unbalanced nitrogen metabolism. By supplementing ornithine upon Arg depletion, both growth and tRNA^Arg ^charging were partially restored.

**Conclusion:**

Under mild stress conditions the basal activity of Gcn2p is sufficient to allow for proper adaptation to amino acid depletion. This study highlights the importance of the GCN2 eIF2 kinase pathway for maintaining metabolic homeostasis, contributing to appropriate tRNA charging and growth adaptation in response to culture conditions deficient for the central amino acids, tryptophan and arginine.

## Background

Translational control is a fundamental step in the regulation of gene expression. An important mechanism for regulating eukaryotic translational initiation involves the phosphorylation of the α subunit of the initiation factor eIF2 in response to environmental stresses [1-3]. The eIF2 delivers initiator tRNA to the translation machinery, and phosphorylation of eIF2α (eIF2α-P) reduces it activity by blocking the eIF2-GDP to eIF2-GTP exchange required for each round of translation initiation. The resulting reduction in global translation conserves energy and allows for cells to reprogram gene expression to ameliorate stress damage. Coincident with this global repression of protein synthesis, eIF2α-P elicits preferential translation of selected mRNAs, including the yeast transcriptional activator *GCN4 *which directs transcription of genes involved in the synthesis of amino acids and the salvaging of nutrients (the so-called general amino acid control, GAAC) [1]. In mammals, a related transcription activator, ATF4, is preferentially translated upon increasing eIF2α-P, contributing to the so-called integrated stress response [4-6].

In the yeast *Saccharomyces cerevisiae*, Gcn2p is the only known kinase for eIF2α phosphorylation [1]. Activation of Gcn2p in response to amino acid starvation occurs by Gcn2p binding to an uncharged tRNA through its histidyl-tRNA-synthetase-like domain [1,7-9]. Although uncharged tRNA^His ^is known to activate Gcn2p *in vivo*, other tRNAs can bind to this HisRS-like domain *in vitro *[8]. We have shown recently that *in vivo*, activation of Gcn2p is coincident with enhanced levels of multiple uncharged tRNAs, and the actual uncharged tRNA activator can vary depending on the type of stress [10]. This genome-wide method for measuring tRNA charging uses selective labeling of tRNAs with Cy3 or Cy5 and microarrays spotted with individual probes for each of the yeast tRNAs. Auxotrophic yeast strains were starved for the limiting amino acid, and the charging levels for both the cognate tRNA and some non-cognate tRNAs were decreased. These key results were confirmed by northern analyses, validating the method and the key findings. These results suggest that activation of Gcn2p occurs in response to a complex pattern of changes in tRNA charging upon starvation for amino acids.

In addition to the GAAC, cells use other mechanisms to regulate the activity of various metabolic pathways. For the aromatic amino acid biosynthetic super-pathway, the first step, biosynthesis of 3-deoxy-darabino-heptulosonate-7-phosphate, is under the control of both *GCN4 *and feedback inhibition of metabolites [11]. While *GCN4 *can upregulate the expression of biosynthetic enzymes, the availability of Tyr and Phe can modulate the enzymatic activity of this step. To control arginine metabolism and the urea cycle, cells elicit transcriptional control by Gcn4p, along with monitoring the intracellular concentration of arginine, ornithine and citrulline to fine tune metabolic flux [12,13]. In this study, we investigated the mechanisms by which yeast cells respond to individual amino acid depletion in a *GCN2 *dependent manner by monitoring cell growth and genome-wide tRNA charging levels. Our results reveal a complex relationship between cellular metabolism and *GCN2 *function.

## Results

### GCN2-dependent recovery of cell growth upon amino acid depletion

**Figure 1:**
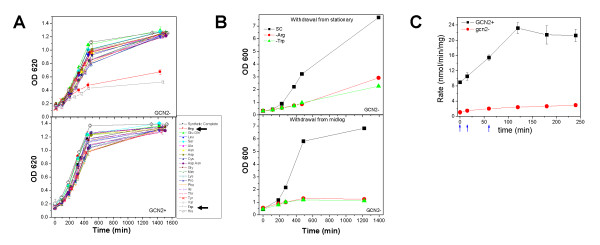
**Yeast growth upon single amino acid depletion shows recovery of Trp and Arg depletion dependent on GCN2**. (A) Systematic depletion of amino acids from growth media in 96 well plates. Arrows indicate slow growth in Arg and Trp in the *gcn2*Δ strain. (B) Recovery of growth in flasks after amino acid depletion. Depletion occurred while cells were in stationary or mid log growth. (C) Measurements of the GCN4-lacZ reporter showed that the Gcn2p-dependent translational regulation of the GCN4 mRNA is significantly reduced in the *gcn2*Δ strain before and after Arg depletion. This result suggests that the level of the GCN4 protein is drastically lower in the *gcn2*Δ strain as expected. Blue arrows indicate time points at which tRNA charging profiles were measured.

Yeast can synthesize each of the twenty amino acids. To screen for conditions in which the *GCN2 *pathway is required for prototrophic yeast cells to recover from amino acid depletion, we systematically depleted one amino acid from the medium for each of the 20 amino acids. Yeast cells were first grown to saturation in SC medium supplemented with all 20 amino acids. Cells were then pelleted and resuspended to A_600 _~ 0.1 in the same medium containing all 20 or just 19 amino acids-absent a selected depleted amino acid. Cell growth was monitored in microplates for 24 h (Fig. 1A). To determine the requirement for *GCN2 *during the nutrient shift, growth of a prototrophic strain containing an intact GAAC pathway was compared to an isogenic *gcn2*Δ strain. The wild-type *GCN2 *strain showed no significant growth differences during these drop-out media conditions. By comparison, the *gcn2*Δ strain showed no growth phenotype upon depletion for each of 18 amino acids; however, depletion of tryptophan or arginine showed a strong growth defect. To address whether depletion for Trp or Arg specifically blocked growth during the transition into early-log growth, we depleted these two amino acids in flasks after cells were grown to the mid-logarithmic phase (Fig. 1B). The same growth defect was observed, indicating that *GCN2 *is required in response to depletion for either Trp or Arg in SC medium. Using a GCN4-lacZ reporter, we show that the Gcn2p dependent translational regulation of the GCN4 mRNA is indeed drastically reduced in the *gcn2*Δ strain before and after depletion of Arg (Fig. 1C, [14]).

### GCN2-dependent tRNA charging effects upon amino acid depletion

**Figure 2:**
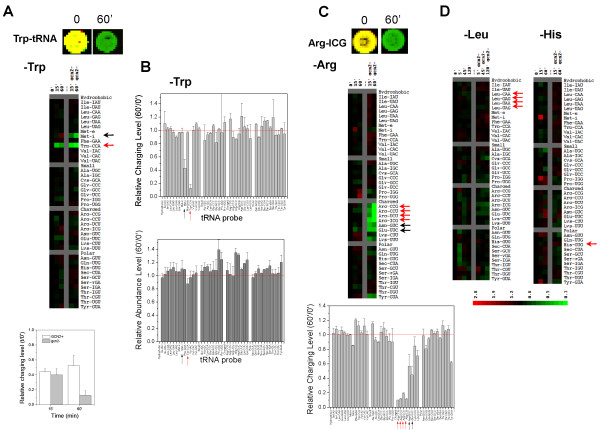
**Changes in tRNA charging profile correlate with growth phenotype**. Cognate tRNAs for the amino acid depleted are indicated by red arrows. Non-cognate tRNAs whose charging also changes significantly are indicated by black arrows. (A) Trp depletion. Top: array spot for the Trp-tRNA probe; Middle: heatmap of the wild-type and isogenic *gcn2*Δ strains; Bottom: histogram of the relative charging level of tRNA^Trp ^before and after Trp depletion. (B) Comparison of the changes in tRNA charging (top) and tRNA abundance (bottom) before and after 60 min of Trp depletion of the *gcn2*Δ strain. Both tRNA^Trp ^and initiator tRNA_i_^Met ^exhibited a large decrease in charging, but not in abundance. (C) Arg depletion. Top: array spot for the Arg-tRNA (anticodon ICG) probe; Middle: heatmap of the wild-type and isogenic *gcn2*Δ strains; Bottom: histogram of the relative charging level before and after 60 min of Arg depletion for the *gcn2*Δ strain. (D) Leu and His depletion showed no significant decrease in tRNA charging.

Since elevated levels of uncharged tRNAs are central to the activation of Gcn2p and the GAAC, we applied a tRNA microarray method to measure the charging level of all tRNAs simultaneously ([10], Fig. 2). Total charged tRNAs were isolated from cells under mildly acidic conditions to retain the amino acid attached to the 3' end. The total RNA sample was then split into two portions, one half was treated with periodate which only oxidizes the 3' end of uncharged tRNAs - this half measures the amount of charged tRNAs. The other half was treated with buffer only - this half measures the amount of total tRNAs. Both halves were deacylated at alkaline pH, and Cy3 and Cy5 fluorophore containing oligonucleotides were ligated onto only those tRNAs with intact 3' ends using T4 DNA ligase. After labeling, samples with opposite fluorophores (e.g. charged tRNA with Cy5 and total tRNA with Cy3) were combined and hybridized on the same microarray.

Both tryptophan and arginine depletion are known to cause growth defects in *gcn4*Δ and the growth rate analysis confirmed that our *gcn2*Δ strain exhibited this phenotype as well ([15-17], Fig. 1). We chose to examine the changes in the tRNA charging profile upon depletion of four amino acids: Trp (Fig. 2A, B) and Arg (Fig. 2C), which showed significant growth defects in the *gcn2*Δ cells, and Leu and His (Fig. 2D), which have extensive but distinct anabolic and catabolic pathways from Trp and Arg. The tRNA charging results showed a significant correlation to the growth profiles for each of these amino acids. Upon Trp depletion, the charging levels of tRNA^Trp ^were lowered by ~2-fold at 15 min for both strains. By 60 min, tRNA^Trp ^charging did not decrease further in the wild-type cells, but declined by 10-fold in the *gcn2*Δ strain (Fig. 2A). This very large decrease of tRNA^Trp ^charging does not correlate with a significant change in the tRNA^Trp ^levels under the same condition (Fig. 2B). Aminoacylation of the initiator tRNA_i_^Met ^was also selectively reduced in the *gcn2*Δ strain in response to removal of Trp. It is unclear why Trp depletion leads to selective decrease in initiator tRNA_i_^Met ^charging. However, reduced charging level of initiator tRNA_i_^Met ^makes biologically sense because this would lead to decreasing the global level of translation and furthermore to up-regulation of GCN4 translation and enhanced GAAC [18]. Upon Arg depletion, the charging levels of all four tRNA^Arg ^isoacceptors were reduced by ~2-fold at 15 min and ~10-fold at 60 min, but only in the *gcn2*Δ strain. The charging of several non-cognate tRNAs, tRNA^Glu ^and tRNA^Asp ^and to a lesser extent tRNA^Lys ^and tRNA^Tyr^, were also lowered in the *gcn2*Δ strain upon Arg depletion in the medium. Therefore, in both cases of Trp and Arg depletion, the growth defect of the *gcn2*Δ strain is accompanied by dramatic charging reductions in cognate tRNAs. The control experiments involving Leu and His depletion showed no appreciable decrease in the charging level of any tRNA in either wild-type or *gcn2*Δ strains, consistent with the null effect of these two amino acid depletions on cell growth.

**Figure 3:**
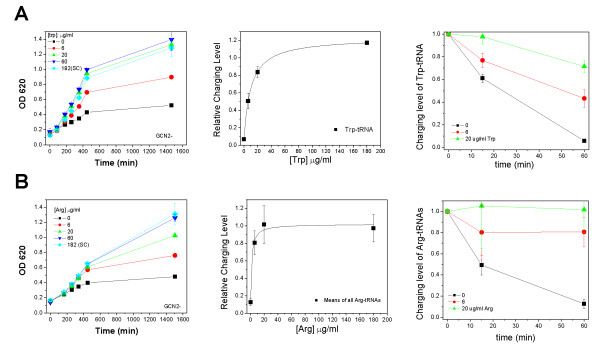
**Partial Trp and Arg depletion of the gcn2Δ strain shows graded sensitivity of Trp and Arg availability to Gcn2p**. The complete media contain 182 μg/ml Trp and 182 μg/ml Arg. (A) Trp. Growth at varying Trp concentrations (left), the tRNA^Trp ^charging levels 60 min after depletion (middle), time course of tRNA^Trp ^charging at various Trp concentration in the media (right). (B) Arg. Growth at varying Arg concentrations (left), the averaged charging levels of all four tRNA^Arg ^isoacceptors 60 min after depletion (middle), time course of the averaged charging levels of all four tRNA^Arg ^isoacceptors at various Arg concentration in the media (right).

To further examine the quantitative dependence and dynamics of tRNA charging upon Trp and Arg depletion in the *gcn2*Δ strain, we examined the effect of partial Trp and Arg depletion in the SC medium (Fig. 3). When all other amino acids are present in the medium, partial Trp depletion showed no change in growth until the Trp concentration was at or below 6 μg/ml (Fig. 3A). tRNA^Trp ^charging levels mirrored this result, as there was no significant decrease in tRNA^Trp ^charging until the Trp concentration was at this same level. The time-dependent drop of tRNA^Trp ^charging was gradual and the time required to reduce tRNA^Trp ^charging by 50% was ~25 min (Fig. 3A). The graded depletion of Arg from the SC medium also showed selective reductions in the growth of the *gcn2*Δ cells, where a threshold of 6 μg/ml elicited a significant reduction compared to the SC medium sated with Arg (Fig. 3B). The tRNA^Arg ^charging levels also reflected this result on growth. The time-dependent drop of tRNA^Arg ^charging was ~10 min for 50% reduction of tRNA^Arg ^charging levels (Fig. 3B). These results indicate that in the absence of *GCN2*, the charging levels of tRNA^Trp ^and tRNA^Arg ^and cell growth are sensitive to the concentration of these two crucial amino acids in the media.

### Cellular pathway linked to Trp sensitivity

**Figure 4:**
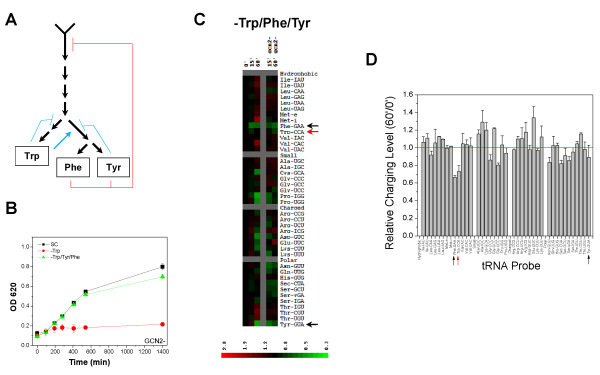
**GCN2-dependent effect upon Trp depletion is linked to aromatic amino acid biosynthesis**. (A) Simplified schematic of aromatic amino acid anabolism indicating where excess amino acids inhibit biosynthesis according to reference [11]. The crucial step is the feedback inhibition of the first step (red lines). (B) Growth curve of *gcn2*Δ with depletion of Trp, Tyr and Phe showing growth recovery in complete media versus Trp depleted media. (C) Heatmap of the wild type and *gcn2*Δ strains depleted of all three aromatic amino acids. Red arrow indicates tRNA^Trp ^and black arrows indicate tRNA^Phe ^and tRNA^Tyr^. (D) Histogram of the relative charging level before and 60 min after triple amino acid depletion for the *gcn2*Δ strain.

In order to better understand why Trp depletion is deleterious to cells lacking *GCN2*, we focused on tryptophan metabolism. Biosynthesis of aromatic amino acids is subject to feedback inhibited and can be activated at multiple steps; however, the most central step for this work is the feedback inhibition at the first reaction of this pathway (Fig. 4A, [19]). This first reaction can be down regulated by an excess of phenylalanine and tyrosine. Additionally, the genes encoding many of these biosynthetic enzymes are transcriptionally regulated by *GCN4*, including *ARO3 *and *ARO4*, which participate in the first step of the pathway leading to the synthesis of chorismate [20,21]. We hypothesized that knocking out *GCN2 *eliminates the ability of the cell to properly regulate aromatic amino acid biosynthesis. When Trp alone was depleted, cellular Trp synthesis cannot proceed normally in the absence of Gcn2p because large amounts of Phe and Tyr are still available in the medium. These two amino acids would then inhibit the first step of this biosynthesis super-pathway, thereby inhibiting the proper synthesis of Trp.

This hypothesis posits that depleting all three aromatic amino acids simultaneously could restore growth in the *gcn2*Δ cells; this was indeed observed (Fig. 4B). The tRNA charging profile after the simultaneous removal of three aromatic amino acids (Fig. 4C, D) showed that restoration of the growth phenotype in the *gcn2*Δ strain is accompanied by the maintenance of high charging levels of tRNA^Trp ^at all times upon Trp/Phe/Tyr depletion, while charging for tRNA^Phe ^and tRNA^Tyr ^showed modest decrease. These results indicate that the requirement for *GCN2 *for growth and maintenance of tRNA charging in the SC medium limiting for Trp is both a consequence of the impaired GAAC and feedback inhibition regulating the carbon flux through the biosynthetic pathway for aromatic amino acids. Depletion of Trp alone retains Tyr and Phe in the SC medium which then can inhibit this combined synthesis pathway. Because Tyr and Phe are readily available in the SC medium depleted of Trp alone, cells should import sufficient amounts of Tyr and Phe to maintain the charging levels of tRNA^Phe ^and tRNA^Tyr^.

### Cellular pathway linked to Arg sensitivity

**Figure 5:**
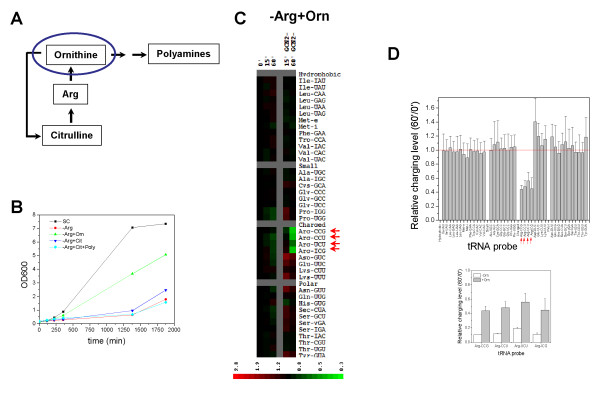
**GCN2-dependent effect upon Arg depletion is linked to nitrogen metabolism**. (A) Simplified schematic of nitrogen metabolism. Ornithine is not only necessary for completion of the urea cycle but is necessary for polyamine biosynthesis. The mitochondrion compartment is shown as a blue oval. (B) Growth curve of *gcn2*Δ strain in complete media, lacking arginine, and lacking arginine supplemented with ornithine, citrulline and/or polyamines. (C) Heatmap of the wild type and *gcn2*Δ strains after depletion of arginine and supplementation with 2 mg/ml ornithine. Arrows indicate tRNA^Arg ^isoacceptors. (D) Histogram of relative charging level before and after Arg-depletion/Orn supplementation for the *gcn2*Δ strain. Inset shows the charging levels of the tRNA^Arg ^isoacceptors in the absence and presence of ornithine.

In order to better understand why Arg depletion is deleterious to cells lacking *GCN2*, we focused on cellular utilization of arginine. Many genes in the arginine biosynthesis are transcriptionally regulated by *GCN4*. Arg is not only important for protein synthesis but is also a necessary precursor for the synthesis of other metabolites. For example, as a part of the urea cycle, Arg is the immediate precursor to ornithine, which is necessary to make citrulline and polyamines (Fig. 5A, [22]). To address the importance of these metabolic relationships in growth phenotype of the *gcn2*Δ cells in Arg-deficient medium, we depleted arginine from the SC media, but supplemented this medium with ornithine or citrulline (Fig. 5B). Growth was partially restored upon ornithine supplementation but not with citrulline. The addition of ornithine also partially alleviated the tRNA^Arg ^charging defect as compared to the absence of ornithine (Fig. 5C, D). Charging of all tRNA^Arg ^isoacceptors was essentially unchanged in the *gcn2*Δ cells after 15 minutes of Arg depletion, and showed a ~2-fold decrease only after 60 minutes, whereas the charging level of tRNA^Arg ^was reduced by 5-10-fold in the absence of ornithine.

Ornithine is only used as a metabolic precursor for citrulline and polyamine biosynthesis. It may be possible that supplementing media with citrulline and polyamines in the absence of arginine would recover growth in a manner similar to ornithine supplementation. However, no growth recovery was observed when citrulline and polyamines were added after arginine depletion (Fig. 5B). Increased concentrations of other nitrogen sources such as Glu and Gln upon Arg depletion also failed to rescue growth (data not shown). These results indicate that aside from Arg, ornithine is unique in this pathway in balancing nitrogen metabolism in the absence of *GCN2*.

## Discussion

The GAAC pathway is essential for regulating metabolism especially when cells are subjected to nutrient stresses. Previous work indicated that activation of Gcn2p in auxotrophic strains produces surprising tRNA charging profiles which can activate Gcn2p and consequently GAAC [10]. In this work we focus on how Gcn2p is used to regulate metabolic flux in prototrophic cells.

While many stresses are known to activate Gcn2p, mild stresses of prototrophic *S. cerevisiae*, such as depleting a single amino acid from the growth media as done in this work do not require activation of Gcn2p under all conditions. One possible reason for this selective response to amino acid availability is that many metabolic pathways contain alternative regulators, in addition to *GCN4 *[23,24]. For example, Chin et al. [25] showed that during the mild nutrient stress of leucine depletion, Leu3p can specially regulated the Leu biosynthetic pathway, and can sufficiently regulate the branched aromatic amino acid biosynthesis super-pathway independent of *GCN4 *up-regulation. Our results agree nicely with this previous study and furthermore, we observed no change in charging of any tRNA upon Leu depletion.

The first step of the aromatic amino acid biosynthetic super-pathway uses feedback inhibition in combination with *GCN4 *for regulation instead of using two separate transcription factors as in Leu biosynthesis. Our results confirm that improper regulation of this super-pathway causes growth and tRNA charging defects in *gcn2*Δ cells subjected to tryptophan depletion. This may be explained by the *gcn2*Δ strain lacking a non-GCN4 protein regulator of the first step of the aromatic amino acid biosynthetic pathway. Lack of Gcn2p mediated up-regulation of *GCN4 *ultimately results in an auxotroph-like phenotype. What would happen to the growth of *gcn2*Δ strain if both Tyr and Phe are depleted? Addition of Tyr and Phe to a yeast strain in which the GCN2 pathway was genetically inactivated did not provide for growth, whereas the addition of Tyr alone or Phe alone did promote growth [15-17]. However, the addition of Trp alone in this yeast strain fully restored growth. These results suggest that as long as Trp is present, double depletion of Tyr and Phe would confer no effect on growth in *gcn2*Δ strains.

Arginine metabolism is also regulated by feedback inhibition of metabolites and by the transcription factor Gcn4p. Unlike tryptophan biosynthesis, arginine metabolism is not strictly a linear pathway. Both arginine anabolism and catabolism are linked to the urea cycle. In the case of the urea cycle, cell uses the available arginine, ornithine, and citrulline to effect enzymatic activity in addition to Gcn4p to regulate metabolic flux [13,26]. While we were unable to completely eliminate the growth defect in *gcn2*Δ strains with supplements, partial growth restoration was possible in media containing high concentrations of ornithine. This result may be related to ornithine transport across cell membranes. Inefficient uptake from the media after arginine depletion could result in disrupted nitrogen flux. Another possibility is that ornithine, a known inhibitor of arginase activity *in vitro *[27], acts as an inhibitor of arginase which converts arginine to ornithine and releases urea as a byproduct. Therefore, it is possible that ornithine may accumulate in the cytosol and inhibit arginase. In this way, the presence of ornithine in the cytosol would reduce the conversion of arginine to ornithine, leaving more arginine available for translation.

We also observed decreased charging levels of specific tRNAs that are not cognate to the depleted amino acid, e.g. initiator tRNA_i_^Met ^upon Trp depletion or tRNA^Asp ^and tRNA^Glu ^upon Arg depletion. In the genetic background used here (*gcn2*Δ), a possible cause of these changes in tRNA charging is derived from regulated expression levels in their cognate aminoacyl-tRNA synthetases prior to amino acid depletion. Transcription of at least one aminoacyl-tRNA synthetase is known to be up-regulated by GCN4 [28]. Hence, the extremely low level of GCN4 protein in the *gcn2*Δ strain (Fig. 1C) could reduce the expression of certain tRNA synthetases, thereby predisposes these cells to become hypersensitive to metabolic fluxes upon amino acid depletion.

## Conclusions

In summary, this work examines growth and genome-wide tRNA charging response in wild type and *gcn2*Δ cells subjected to mild nutritional stress. Although eIF2 phosphorylation was thought to control translation upon the depletion of any amino acid, we found that this conserved mechanism of translational control is particularly sensitive to the availability of just two amino acids, Trp and Arg. This GCN2 dependent response to the limitation of Trp and Arg is due to synthesis of Trp and degradation of Arg. Our results show unexpected complexity of cellular metabolism and tRNA charging, and indicate that some metabolic pathways are particularly sensitive to translational control.

## Methods

### Cell strains and growth

The *S. cerevisiae *strains used in this study were derived from EG328-1A (*MAT*α *ura3-52 leu2 trp1*) [29,30]. Strain EG328-1A was transformed with DNA fragments containing the *LEU2*, *TRP1*, or *URA3 *genes, generating a prototrophic strain, WY798 (*MAT*α *URA3 LEU2 TRP1*). An isogenic strainWY799 (*MAT*α *gcn2::URA3 LEU2 TRP1*) contains a deletion in the G*CN2 *gene. Cells were grown overnight in filter sterilized synthetic complete (SC) media containing 2% dextrose, 0.5% ammonium sulfate, and all amino acids [31]. To screen for growth in media with selected amino acids, cells were grown overnight to saturation (A_600 _~ 6-7) in SC media containing all amino acids. Cells were collected by centrifugation, and then resuspended in SC media lacking only the specified amino acid to A_600 _of ~0.1. Cells were grown in 96 well plates at 30°C with constant shaking. For flask growth an overnight culture of cells was diluted down to A_600 _of 0.1 and grown in SC media until an A_600 _of 0.4-0.5. Cells were pelleted and then resuspended in SC media lacking only the specified amino acid and growth was monitored for up to 24 h.

### GCN4-lacZ reporter assay

WY523 and WY525 (*gcn2Δ*) cells were derived from strain EG328-1A transformed with plasmid p180, encoding GCN4-lacZ with the full complement of upstream ORFs [14]. Cells were grown overnight in SC media containing all amino acids. Cells were diluted down to A_600 _~ 0.1, then grown to A_600 _~ 0.4-0.5 at which point they were spun down and resuspended in media lacking the specified amino acid. 35 ml cells were taken at designated time points and lysed using beads in 0.4 ml of 100 mM Tris-HCl pH 8, 20% glycerol, 1 mM β -mercaptoethanol and 100 μM PMSF. Lysate was clarified with a 10 min spin at 18,600 RFC. 20 μl of extract was combined with 980 μl of reaction buffer (100 mM Sodium Phosphate, pH 7.5, 10 mM KCl, 2 mM MgSO_4_, 4.5 mM β-mercaptoethanol). 200 μl of o-nitrophenyl-β-D-galactopyranoside solution (4 mg/ml) was added and the reaction was allowed to proceed for 20 and 60 min at 28°C. Reaction was quenched by adding 0.5 ml of 1 M Na_2_CO_3 _and absorbance was taken at 420 nm.

### Genome-wide tRNA charging profile by microarrays

Isolation, genome-wide profiling of charged tRNA was carried out as previously described [10] and in a Method video article [32]. The microarray data has been deposited in Geo data base (accession number GSE22452).

### Genome-wide tRNA abundance profile by microarrays

the detailed procedures used for profiling tRNA abundance have been described previously [33,34].

## List of abbreviations

GAAC: general amino acid control.

## Authors' contributions

JZ carried out the growth studies and microarray analysis. XCW assisted with the growth studies and performed some microarray analysis. RW participated in design of the study. TP participated in the design of the study and the preparation of the manuscript. All authors read and approved the final manuscript.
